# Production of isoform-specific knockdown/knockout Madin–Darby canine kidney epithelial cells using CRISPR/Cas9

**DOI:** 10.1016/j.mex.2020.101149

**Published:** 2020-11-17

**Authors:** James M. Readler, Amal S. AlKahlout, Abimbola O. Kolawole, Katherine J.D.A. Excoffon

**Affiliations:** a Biomedical Sciences PhD Program, Wright State University, Dayton, OH 45435, United States; b Department of Biological Sciences, Wright State University, 3640 Colonel Glenn Hwy, 235a BS, Dayton, OH 45435, United States; c Boonshoft School of Medicine, Wright State University, Dayton, OH 45435, United States

**Keywords:** CRISPR-Cas9 editing, Coxsackievirus and adenovirus receptor (CAR), Madin–Darby canine kidney epithelial (MDCK) cells, Polarized epithelium

## Abstract

CRISPR-Cas9 gene editing has made it possible to specifically edit genes in a myriad of target cells. Here, a method for isoform-specific editing and clonal selection in Madin-Darby canine kidney (MDCK) epithelial cells is described in detail. This approach was used to address a long-standing question in virology of how adenovirus enters polarized epithelia from the apical surface. Our method relies on selecting two sgRNA sequences, cloning them into a suitable fluorescently labeled Cas9 vector system, and subsequently transfecting our MDCK epithelium and selecting isoform-specific Coxsackievirus and adenovirus receptor knockout clones. Utilization of this method is readily applicable to many other genetic targets in epithelial cells.

• Simultaneous utilization of an sgRNA upstream and an sgRNA downstream of a target sequence allows for deletion of the intervening sequence, including whole exons.
• Sorting of cells positive for fluorescent marker gene expression enhances the identification of partial and biallelic gene knockout.
• PCR screening allows relatively fast and efficient determination of isoform-specific deletion.

## Graphical abstract

**Figure:**
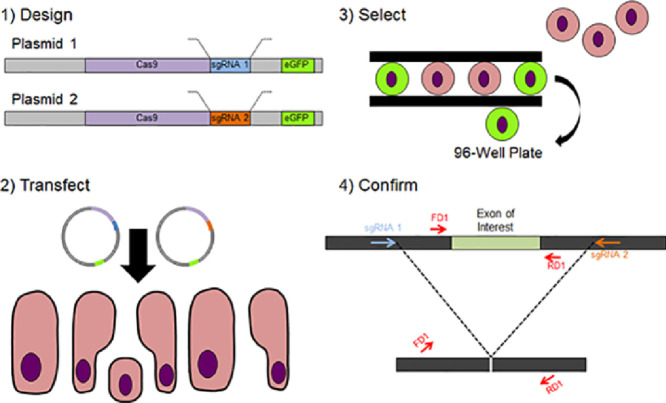

Specifications Table

**Table:** 

Subject Area:	Biochemistry, Genetics and Molecular Biology
More specific subject area:	Molecular biology, gene editing
Method name:	Production of isoform-specific knockdown/knockout epithelial cell lines
Name and reference of original method:	Isoform specific editing of the coxsackievirus and adenovirus receptor. Readler JM, AlKahlout AS, Sharma P, Excoffon KJDA. Virology. 2019 Oct;536:20–26. doi: 10.1016/j.virol.2019.07.018. PMID: 31,394,408
Resource availability:	www.blast.ncbi.nlm.nih.gov/Blast.cgi www.genome.jp/tools-bin/clustalw www.chopchop.cbu.uib.no takarabio.com/learning-centers/cloning/in-fusion-cloning-tools

## Method details

### Reagents

• ATCC CCL-34 MDCK cell line modified as described in Kotha et al. 2015 [1]
• pspCas9(BB)−2A-GFP plasmid (PX458; Addgene, Watertown, MA)
• sgRNA primers (Integrated DNA Technologies, Coralville, IA)
• Infusion Cloning kit (Cat# 638,916; Takara Bio USA, Mountain View, CA)
• CloneAmp Hifi PCR Premix (Cat# 639,298; Takara Bio USA, Mountain View, CA)
• DNA Clean & Concentrator (Cat# D4003; Zymo Research, Irvine, CA)
• Stellar competent cells (Cat # 636,766; Takara Bio USA, Mountain View, CA)
• Standard Taq Polymerase (Cat# MS0273X; New England Biolabs, Ipswich, MA)
• Dehydrated Culture Media: LB Broth, Miller (Luria-Bertani) (Cat# 244,610; BD Difco, Franklin Lakes, NJ)
• Ampicillin (Cat# 69–52–3; ThermoFisher, Waltham MA)
• Agar (Cat# J637; Ameresco, Framingham, MA)
• QIAprep Spin Miniprep Kit (Cat# 27,104; Qiagen, Hilden Germany)
• EndoFree Plasmid Maxi Kit (Cat# 12,362; Qiagen, Hilden, Germany)
• DNeasy Blood and Tissue kit (Cat# 69,506; Qiagen, Hilden, Germany)
• DreamFect Gold transfection reagent (Cat# DG81000; OZ Biosciences, San Diego, CA)
• TrypLE Express (Cat# 12,604–013; ThermoFisher-Gibco, Waltham, MA)
• Phosphate buffered saline without calcium or magnesium (PBS-/-) (Cat# SH3002.02; GE Healthcare Life Sciences-Hyclone, Marlborough, MA)
• Minimum Essential Medium (MEM) (Cat# 50–010-PB; Corning Life Sciences, Corning, NY)
• Fetal Bovine Serum without tetracycline (Cat# S10350; Atlanta Biologicals, Flowery Branch, GA)
• Penicillin/Streptomycin (Cat# 15,140–122; ThermoFisher- Gibco, Waltham MA)
• Dimethyl Sulfoxide (DMSO) (Cat# N182; Ameresco, Framingham, MA,)
• Sodium Bicarbonate (Cat# 0865; Ameresco, Framingham, MA)
• EDTA (0.5 M) (Cat# 15,575–038; ThermoFisher- Gibco, Waltham MA)
• HEPES (1 M) (Cat# 15,630–080; ThermoFisher- Gibco, Waltham MA)
• Opti-MEM (Cat# 11,058–021; ThermoFisher- Gibco, Waltham MA)
• 500 mL capacity 0.2 µm filter units (Cat# FB12566504; Fisher Scientific, Waltham, MA)
• 0.4% Trypan Blue (Cat# 15,250; ThermoFisher-Gibco, Waltham MA)
• Trypsin-EDTA (0.25%), phenol red (Cat# 25,200,056; ThermoFisher-Gibco, Waltham, MA)

### Recipes

• *Luria broth:* 25 g Luria broth powder (Cat# 244,610; BD Difco, Franklin Lakes, NJ) per 1 L of milli Q purified water. +/- 50 mg/mL ampicillin (Cat # 69–52–3; ThermoFisher, Waltham MA).
• *Luria broth plates:* 12 g agar (Cat# J637; Ameresco, Framingham, MA) per 1 L of Luria broth (BD Difco, Franklin Lakes, NJ). +/- 50 mg/mL ampicillin (Cat # 69–52–3; ThermoFisher, Waltham MA).
• *MDCK media*: 4.77 g MEM powder (Cat# 50–010-PB; Corning Life Sciences, Corning, NY) dissolved in 500 mL milli Q purified water + 1.1 g sodium bicarbonate (Cat# 0865; Ameresco, Framingham, MA). Titrate to physiological pH (7.35–7.45) and then add 5% fetal bovine serum (FBS) without tetracycline (Cat# S10350; Atlanta Biologicals, Flowery Branch, GA) and 1% penicillin/streptomycin (Cat# 15,140–122; ThermoFisher- Gibco, Waltham MA). Filter sterilize with 500 mL capacity 0.2 µm filter units (Cat# FB12566504; Fisher Scientific, Waltham, MA).
• *Fluorescence activated cell sorting (FACS) buffer:* 1 mM EDTA (Cat# 15,575–038; ThermoFisher- Gibco, Waltham MA), 25 mM HEPES (Cat# 15,630–080; ThermoFisher- Gibco, Waltham MA) and 1% FBS (Cat# S10350; Atlanta Biologicals, Flowery Branch, GA) in PBS-/- (Cat# SH3002.02; GE Healthcare Life Sciences-Hyclone, Marlborough, MA). Filter sterilize with 0.22 µm syringe filter (Cat# F-2690–7, Intermountain Scientific Corporation, Kaysville, UT).

### Equipment

• Biosafety cabinet
• 37 °C incubated orbital shaker
• 37 °C humidified incubator with 5% CO_2_
• Micropipettors and tips
• Multichannel micropipettor
• Pipet aid with 5, 10, and 25 ml serological pipettes
• 500 mL capacity 0.2 µm filter units
• 0.22 µm syringe filters
• 1.5 ml microfuge tubes
• Table top microtube centrifuge, 4  °C and room temperature (RT)
• 50 °C heating block
• Nanodrop 2000 (ThermoFisher, Waltham MA)
• Olympus IX83 Microscope (Olympus Corporation, Tokyo, Japan)
• Olympus DP74 Microscope Digital Camera (Olympus Corporation, Tokyo, Japan)
• Olympus cellSens software (Olympus Corporation, Tokyo, Japan)
• Countess II FL Automated Cell Counter (Thermofisher, Waltham, MA)
• Nunc Lab-Tek 4-well Chamber Slide with removable wells (Cat# 154,526; ThemoFisher, Waltham, MA)
• Cytoone 24-well plates (Cat# CC7682–7524; USA Scientific, Ocala, FL)
• Photoshop Software
• Becton Dickinson FACSAria III system (Franklin Lakes, NJ)
• Computer and internet access
• Mastercycler nexus eco (Eppendorf, Hamburg, Germany)
• Milli Q water purification system (EMD Millipore, Burlington, MA)

### Procedure

Step 1: Selection of the single guide RNA sequences (sgRNAs).

(1) Obtain the target exon nucleotide sequence along with the surrounding intronic sequence using the NCBI consensus sequence available for *Canis lupus familiaris* (dog) at www.ncbi.nlm.nih.gov/genome/.
(2) Validate that this sequence is consistent in the cell line of interest by using candidate primers (e.g. IDT Primer Quest tool) to PCR amplify the associated segments of target cell genomic DNA, isolated via Qiagen DNeasy Blood and Tissue Kit (Hilden, Germany) according to the manufacturer's instructions, and subjecting the amplicons to Sanger sequencing. Note that a PCR purification step with a DNA Clean & Concentrator (Zymo Research, Irvine, CA) may be necessary for sequencing. Sequence alignment can be performed with NCBI nucleotide Blast (blast.ncbi.nlm.nih.gov/Blast.cgi) or the freely available Clustalw multiple alignment program (www.genome.jp/tools-bin/clustalw).
(3) Import the validated sequence of your genetic target into the CHOP-CHOP software (chopchop.cbu.uib.no, Harvard University) and select the species from which the target cell line is derived.
(4) Use the CHOP-CHOP software to generate a ranked list of potential single guide RNAs (sgRNAs) and select at least one upstream of the target sequence and one downstream of the target sequence. General considerations for sgRNA selection are discussed below.

Step 2: Cloning sgRNAs into suitable Cas9 expression plasmids.

(1) According to the manufacturer's instruction for Infusion cloning, design primers containing the sgRNA sequence flanked at the 3′ end with 23–25 nt of sequence homologous to the plasmid vector. Generate primers using the Infusion Cloning Primer Design Tool (takarabio.com/learning-centers/cloning/in-fusion-cloning-tools, Takara Bio USA).
(2) Synthesize primers using Integrated DNA Technologies (Coralville, IA) and use them in a 20 µL inverse PCR reaction, with CloneAmp Hifi PCR Premix (Takara Bio USA, Mountain View, CA) The product of this inverse PCR reaction is linearized pspCas9(BB)−2A-GFP plasmid (Addgene, Watertown, MA) and contains the sgRNA sequence of interest in frame with the Cas9 gene.
(3) Treat 5 µL of the PCR reactions with 2 µL of the cloning enhancer reagent provided in the kit in a thermocycler for 15 min at 37 °C and then 15 min at 80 °C.
(4) Mix the resulting product with 5X infusion cloning buffer and dilute to 1X with molecular biology grade water, then incubate at 50 °C for 15 min.
(5) Transform the resulting product into stellar competent cells (Takara Bio USA, Mountain View, CA) at a ratio of 2.5 µL of reaction product to 50 µL competent cells. Plate transformed cells onto Luria broth plates containing 50 mg/mL ampicillin and incubate at 37 °C overnight.
(6) Choose 5 colonies to grow in Luria broth containing 50 mg/mL ampicillin at 37 °C overnight. Also, spot colonies to a Luria broth plate with 50 mg/ml ampicillin, grow overnight at 37 °C and keep at 4 °C for future expansion.
(7) Isolate plasmids from Luria broth cultures using a QIAprep Spin Miniprep Kit (Qiagen, Hilden Germany). A plasmid map is included in Supplemental Figure 1.
(8) Perform a Sanger sequencing reaction of plasmids with a U6 universal primer to ensure proper integration of the sgRNA molecule in-frame with the Cas9 gene (supplemental Table 1).
(9) Expand two plasmids with confirmed sequences, one which recognizes a sequence upstream of the target and one that recognizes a sequence downstream of the target, by inoculating 250 mL of Luria broth containing 50 mg/ml ampicillin and growing overnight in a 37 °C shaker.
(10) Isolate purified plasmid using an EndoFree Plasmid Maxi Kit (Qiagen, Hilden, Germany). Quantify plasmid concentration using a Nanodrop 2000 (ThermoFisher, Waltham MA).

Step 3: Generation and expansion of potential knockout (KO) clones.

(1) Seed MDCK cells at 2.4 × 10^4^ cells per well in 24-well plates and grow overnight in MDCK media at 37 °C in a humidified incubator with 5% CO_2_. Seeding at this density allows the cells to be approximately 70% confluent at the time of transfection.
(2) The following day, double transfect cells with a pspCas9(BB)−2A-GFP plasmid (Addgene, Mountain View, CA) encoding an sgRNA that recognizes intronic sequence upstream of *CXADR* Exon 8 and one that encodes an sgRNA that recognizes intronic sequence downstream of *CXADR* Exon 8. *CXADR* Exon 8 was chosen because it encodes the C-terminus of the 8-exon encoded isoform of the Coxsackievirus and adenovirus receptor, CAR^Ex8^, an isoform of the Coxsackievirus and adenovirus receptor (CAR) that can localize to the apical surface of airway epithelia and promote apical adenovirus infection [1], [2], [3]. Use DreamFect Gold transfection reagent (OZ Biosciences, San Diego, CA) according to the manufacturer's instructions. Briefly, mix 250 ng of the upstream plasmid and 250 ng of the downstream plasmid (500 ng total DNA) with 2 µL of DreamFect Gold transfection reagent (OZ Biosciences, San Diego, CA) in 100 µL OptiMEM. Then add this mixture dropwise to MDCK cells, already containing 400 µL of MDCK complete media, and incubate for 48 h at 37 °C in a humidified incubator with 5% CO_2_.
(3) After incubation, confirm transfection by checking for the presence of GFP positive cells using a fluorescence microscope.
(4) Lift MDCK cells using TrypLE Express (Gibco, Gaithersburg, MD) and pellet by centrifugation at 150 x *g* for 5 min.
(5) Reconstitute the MDCK cell pellet was in fluorescence activated cell sorting (FACS) buffer and sort GFP positive cells using a Becton Dickinson FACSAria III system (Franklin Lakes, NJ) such that 1 GFP positive cell enters 1 well of a 96-well plate.
(6) Incubate single MDCK cell cultures at 37 °C in a humidified incubator with 5% CO_2_, changing MDCK media with a multi-channel micropipettor every 3 days in order to minimize the chance of washing single cells off the plate.
(7) As colonies reach confluence, lift with TrypLE (Gibco, Gaithersburg, MD), pellet by centrifugation at 150 x *g* for 5 min, and expand into single wells of 24-well plates.
(8) Once sufficiently expanded, isolate genomic DNA from a portion of the cells from each clone using a Qiagen DNeasy Blood and Tissue kit (Hilden, Germany). Continue to expand the rest of the cells until knockout clones can be identified as described below.

Step 4 Screening and validation of potential knockout clones.

(1) Analyze Genomic DNA of potential KO clones by first performing a screening PCR using primer pairs inside the region targeted for deletion with Standard Taq Polymerase (New England Biolabs, Ipswich, MA) using cycling parameters optimized by the manufacturer. The primer sequences that recognized nucleotides within the deleted region used for our JR1-CAR^Ex8^-KO cell line were F-GACCCATAAGGGAAGCCTAAC and R-ATGCCTGGTGCCACTTTAT [2].
(2) Subject clones that do not produce an amplicon in the screening PCR to an additional PCR reaction using primers that recognized sequence outside of the expected deletion site. The presence of amplicon size shifts between the parental cell line and potential KO line indicates that DNA deletion events have occurred [2,4].
(3) Knockout/knockdown phenotypes can then be validated using a variety of assays. In our case, we measured isoform specific protein expression of CAR and differences in adenoviral transduction [2].
(4) Freeze down aliquots of chosen cells in complete MDCK media with 5% DMSO first at −80 °C prior to being moved to a liquid nitrogen tank for long-term storage. Of note, different cell lines may require more conservative freezing parameters, thus, it is recommended that labs use freezing protocols that have been optimized for their cell line of interest.

## Method validation

Step 1: Selection of the single guide RNA sequences (sgRNAs).

Simultaneous utilization of two 20 nucleotide sgRNAs, one upstream of the target sequence and one downstream of the target sequence, has previously been described to allow for deletion of the intervening segment [4,5]. The rate of diploid deletion events is dependent upon the distance between double stranded breaks with, shorter distances being more efficient [5]. The intronic and exonic sequence for MDCK cells can be obtained from the NCBI canine genome sequence. When designing sgRNA's that recognize intronic sequence, it is important to determine the sequence in the individual cell line that will be used for editing, as discrepancies were noticed between the NCBI sequence and genome of the MDCK cell line used in these experiments. We accomplished this by generating candidate primers, PCR amplifying the regions surrounding the 8th exon of the *CXADR* gene, and, after PCR product purification (DNA Clean & Concentrator, Zymo Research, Irvine, CA), subjecting these amplicons to commercial Sanger sequencing (Genewiz, South Plainfield, NJ).

Target gene sequences can be entered into the freely available CHOP-CHOP software (Harvard University) to generate candidate sgRNA sequences and rank them based upon predicted cutting efficiency and minimal predicted off target effects [6,7]. It is generally considered that sgRNA sequences should be at least 3 nucleotides different than any other sequence present in the genome of the target cell [8]. Furthermore, a guanine nucleotide in the sgRNA immediately adjacent to the PAM sequence has been reported to increase cutting efficiency [9]. We incorporated the sequencing results for the *CXADR* exon 8 region in our MDCK cell line into the CHOP-CHOP software under the *Canis Lupis Familiaris* filter and selected a suitable sgRNA that targets intronic sequence upstream of *CXADR* exon 8 (CGAAGGGCAAAATCTTCTAG) and one that targets intronic sequence downstream *CXADR* exon 8 (GGTTGCCTTGGGGAAAGTTA).

Step 2: Cloning sgRNAs into suitable Cas9 expression plasmids.

In order for proper expression of Cas9/sgRNA complexes and selection of transfected cells, a suitable Cas9 expression plasmid must be selected. For sgRNA sequences designed for use in MDCK cells, an Infusion Cloning kit (Takara Bio USA, Mountain View, CA) was used to incorporate sgRNA sequences into the pspCas9(BB)−2A-GFP plasmid (Addgene, Watertown, MA). The main advantage of the pspCas9(BB)−2A-GFP plasmid is that transfected cells turn green, thus making them amenable to FACS to allow enrichment of transfected cells and isolation of potentially edited clonal populations [10]. While this plasmid contains BbSI sites that can allow incorporation of candidate sgRNA sequences using more traditional cloning techniques, performing site directed mutagenesis via inverse PCR with an Infusion cloning kit is another highly efficient viable option for sgRNA integration [10,11].

Step 3: Generation and expansion of potential knockout clones.

**Fig. 1:**
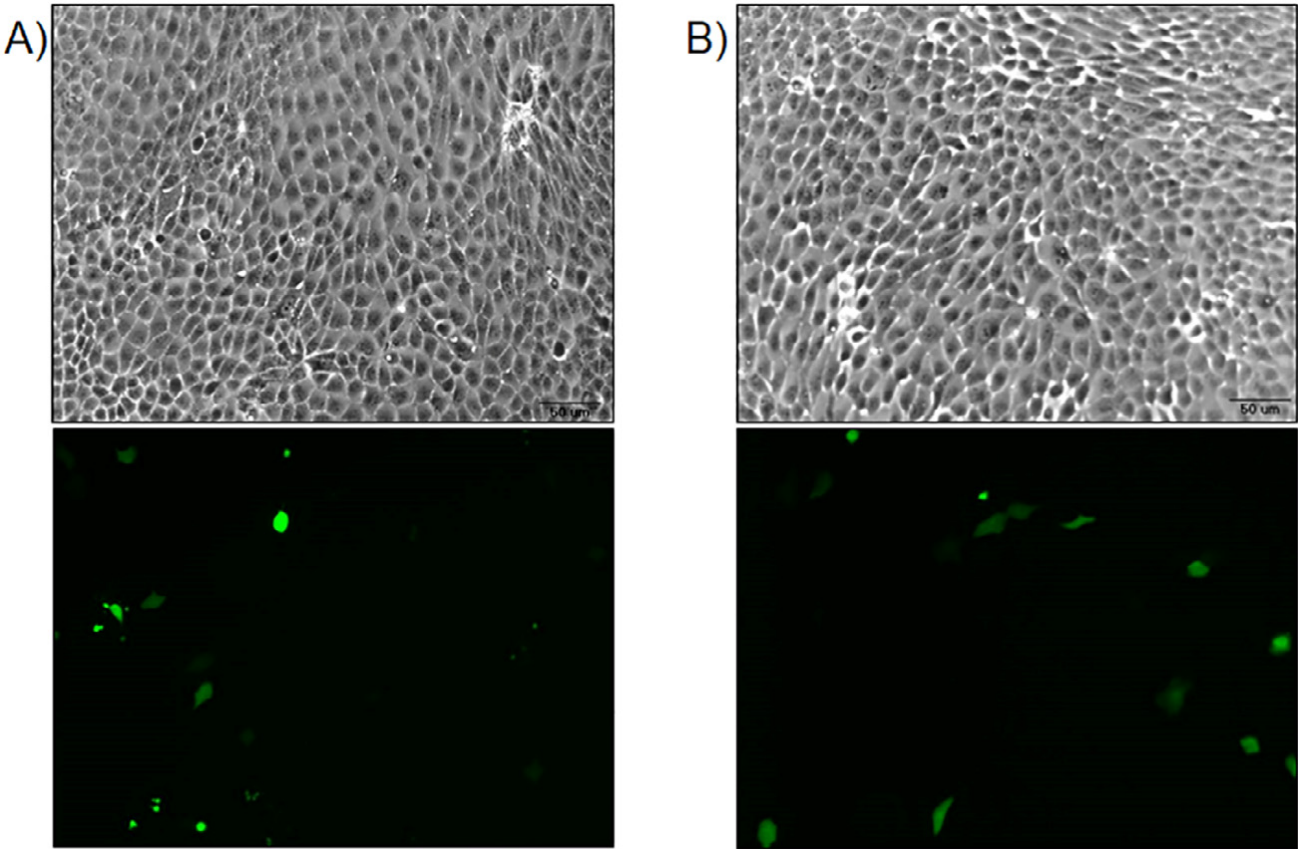
pspCas9(BB)−2A-GFP plasmids transfection into MDCK cells. Fluorescence microscopy depicting brightfield (top) and corresponding GFP (bottom) images of MDCK cells transfected with pspCas9(BB)−2A-GFP plasmids encoding sgRNAs that A) recognize intronic targets upstream and B) downstream of *CXADR* exon 8. Images were taken on an Olympus IX83 microscope with an Olympus DP74 microscope digital camera using Olympus cellSens software. Images were taken at 10X magnification. Brightness and contrast of images were adjusted using Photoshop software. Black bars represent 50 µm.

After successful production of Cas9 plasmids with properly inserted sgRNA sequences, plasmids must be delivered into the cell line of interest and transfected cells must be isolated for expansion and analysis. We double transfected plasmids containing the sgRNA that recognizes intronic sequences upstream or downstream of *CXADR* exon 8 into our MDCK cell line using DreamFect Gold transfection reagent (OZ Biosciences, San Diego, CA). Transfection of either plasmid alone resulted in a transfection efficiency of approximately 10–15% (Fig. 1). Two days later, this cell population was subjected to FACS sorting into a 96-well plate, such that a single GFP positive cell was present per well.

Clonal populations were expanded in culture for approximately 2 weeks until wells were near confluence. Despite being careful to ensure adequate nutrition and minimizing the risk of washing single cells off the plate by changing growth media every 3 days, we ultimately expanded 14 clonal populations from a single plate starting with 96 potentially edited cells. Thus, it is important to sort a large number of cells in order to generate a modest number of clonal populations. Furthermore, although not strictly necessary, changing media with a multichannel pipettor speeds up the process and minimizes the time that cells are without media.

Step 4 Screening and validation of potential knockout clones.

**Fig. 2:**
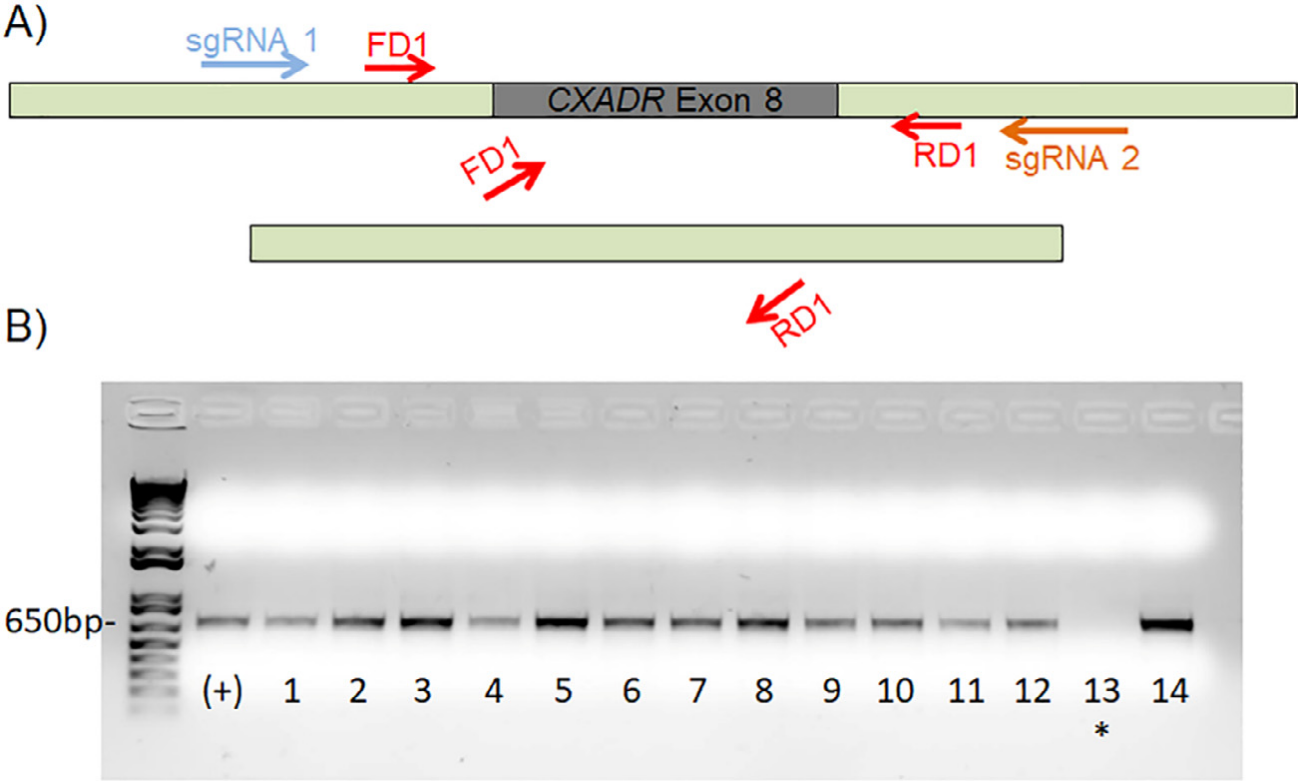
PCR screening identifies clone 13 as a possible genomic CAR^Ex8^ knockout clone. A) Schematic representation of the PCR reaction with primers within the region expected to be deleted (PCR 1). If the sequence between the sgRNA that recognizes intronic sequence upstream of CXADR exon 8 (blue arrow) and the sgRNA that recognizes sequence downstream of CXADR exon 8 (orange arrow) is deleted, primers that bind sequence within the deleted segment (red arrows) will be unable to bind and there will be no amplification in the forward (FD) or reverse (RD) direction. B) Deletion PCR reactions for potential CAR^Ex8^ KO clones (numbers) and MDCK parental cells (+) run on an agarose gel. Clone 13 (marked by asterisk) did not amplify, indicating the possibility of a deletion event.

**Fig. 3:**
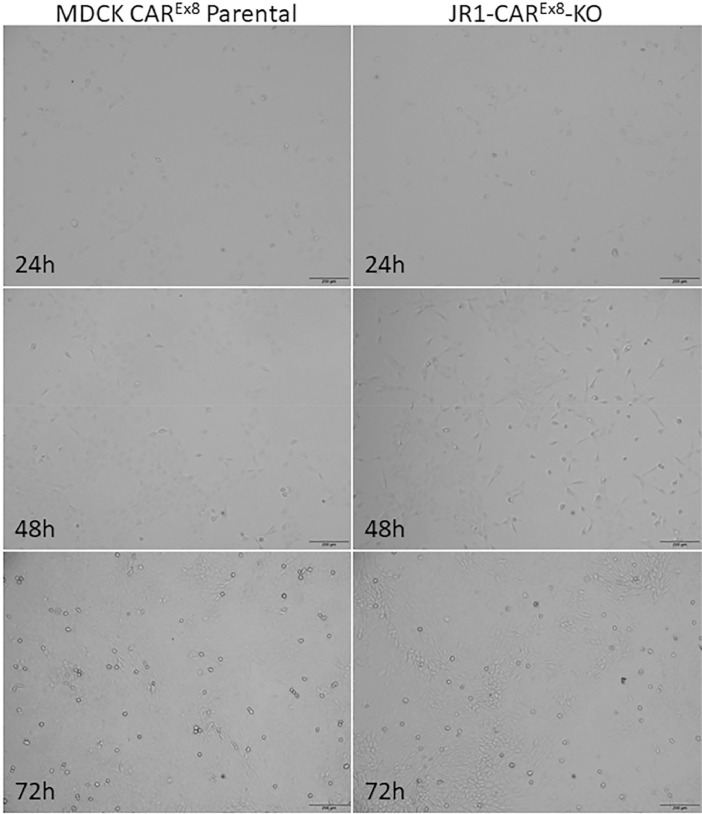
Parental MDCK cells and JR1-CAR^Ex8^-KO MDCK exhibit similar morphology. Parental MDCK cells (left) and JR1-CAR^Ex8^-KO cells (right) were seeded on chamber slides at similar concentrations. Bright field microscopy images were taken at 24, 48, and 72 h post seeding. Images were taken on an Olympus IX83 microscope with an Olympus DP74 microscope digital camera using Olympus cellSens software. Images were taken at 10X magnification. Black bars represent 200 µm.

**Fig. 4:**
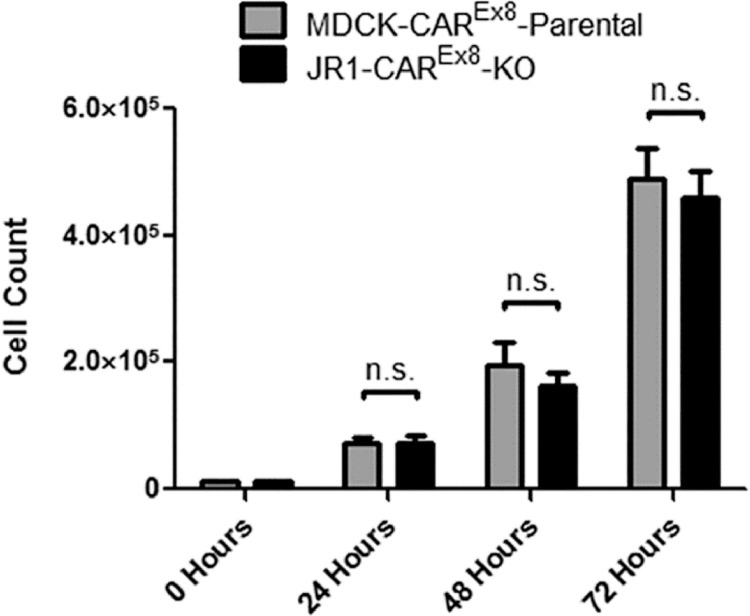
Parental MDCK cells and JR1-CAR^Ex8^-KO MDCK cells exhibit similar growth characteristics. Parental MDCK cells (gray) and JR1-CAR^Ex8^-KO cells (black) were seeded in 24-well plates at similar concentrations and then counted at 24, 48, and 72 h post seeding. Data are pooled from 3 independent experiments, each with *n* = 4 per time point. Error bars represent standard error of the mean. Two-tailed T-test comparison of Parental MDCK cells and JR1-CAR^Ex8^-KO MDCK at each individual time point was not statistically significant (*p* = 0.655 at 24 h, *p* = 0.089 at 48 h, and *p* = 0.524 at 72 h).

Once DNA is isolated from clonal populations of CRISPR/Cas9 treated cells, screening and validation of the gene-edited phenotype can be performed. It is important to note that cells were selected based upon successful transfection (GFP positivity) and may or may not have undergone gene editing. When Cas9 mediated double stranded breaks occur, there are many potential genetic consequences, such as the incorporation of random insertions and deletions (indels) at the cut site as a result of nonhomolgous DNA repair or reannealing of the broken strand via homologous end joining [12]. Many techniques have been developed to assess the presence of genetic editing [13]. In our method, we wanted to select for cells that had two simultaneous double-stranded breaks on both alleles, one upstream of *CXADR* exon 8 and one downstream of *CXADR* exon 8, and deletion of the intervening segment of DNA. In order to screen for such cells, we subjected genomic DNA from each clonal population to two screening PCRs, one with primers that recognize sequence inside of the deleted region (PCR 1) and one that recognize sequence outside of deleted region (PCR 2). This technique allows for the identification and selection of cells that underwent diploid deletion, haploid deletion, or no deletion events [4,5]. Of the 14 clones obtained from the first round, one clone (Clone 13) did not amplify a 650 bp band during PCR 1 indicating that it might be a double deletion clone (Fig. 2). This clone and several others were subjected to PCR 2 and a band shift from approximately 1.5 kb in the control cells to 300 bp in clone 13 was seen, suggesting biallelic deletion of the intervening segment containing *CXADR* exon 8 [2]. This clone was then referred to as JR1-CAR^Ex8^-KO. Cell morphology and division rates were then compared between JR1-CAR^Ex8^-KO epithelial cells and their parental counterparts. The same number of cells (1.2 × 10^4^ cells/well) were seeded in either 24-well plates, for counting, or 4-well chamber slides, for microscopy, and grown for 24, 48, and 72 h in a 37 °C humidified incubator with 5% CO_2_. Representative bright field images were taken at 24, 48, and 72 h post seeding using an Olympus IX83 microscope with an Olympus DP74 microscope digital camera using Olympus cellSens software (Fig. 3). Cells were counted by first lifting with trypsin and then diluting into 1 mL of media. This cell suspension was pipetted vigorously to ensure all cells were lifted and then 10 µL of cell suspension was mixed 1:1 with 0.4% Trypan blue. This mixture was then counted using a Countess II Automated Cell Counter (Fig. 4).

After utilization of screening PCRs to select for double deletion clones, knockout/knockdown of the gene should be confirmed. A variety of techniques can be used for this, including total mRNA isolation and subsequent reverse transcriptase qPCR to measure gene expression and/or methods such as Western blotting or immunohistochemistry to identify expression of the protein of interest. In our case JR1-CAR^Ex8^-KO cells were subjected to Western blot with two primary antibodies, one that detects total CAR and one that specifically detects CAR^Ex8^, and it was found that cells had drastic reduction in CAR^Ex8^ expression with only a modest reduction in total CAR expression, consistent with isoform specific knockdown of CAR^Ex8^ [2].

## Conclusions

The method outlined in this manuscript describes how to use CRISPR/Cas9 technology to delete sections of genomic DNA in MDCK epithelial cells. The deleted segment can be of variable length and if it contains target exons, can result in isoform specific deletion of genes. It is recommended that multiple knockout clones be obtained for any one gene in order to confirm that the resulting phenotype is due to target gene knockout and not potential off-target effects. This method was used to create a virus receptor isoform-specific knockdown cell line by deleting the 8th exon of the *CXADR* gene. This method is expected to be readily amenable to other genetic targets and to other epithelial cell lines capable of being expanded from single cells.

## Declaration of Competing Interest: [MANDATORY – Delete as appropriate]

X The authors declare that they have no known competing financial interests or personal relationships that could have appeared to influence the work reported in this paper.

## Appendix Supplementary materials

Image, application 1
