# Supplementary material for: Production of isoform-specific knockdown/knockout Madin–Darby canine kidney epithelial cells using CRISPR/Cas9
Source: MethodsX. 2020 Nov 17;7:101149. doi: 10.1016/j.mex.2020.101149 (PMC7718483; doi:10.1016/j.mex.2020.101149)
Supplement: Supplementary file 1 [file mmc1.docx]

**Supplementary material *and/or* Additional information:**


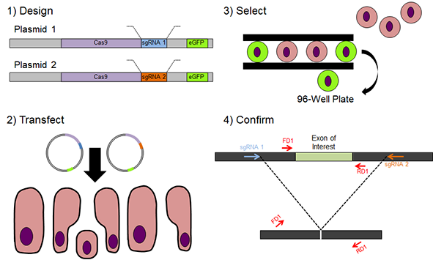


Graphical Abstract


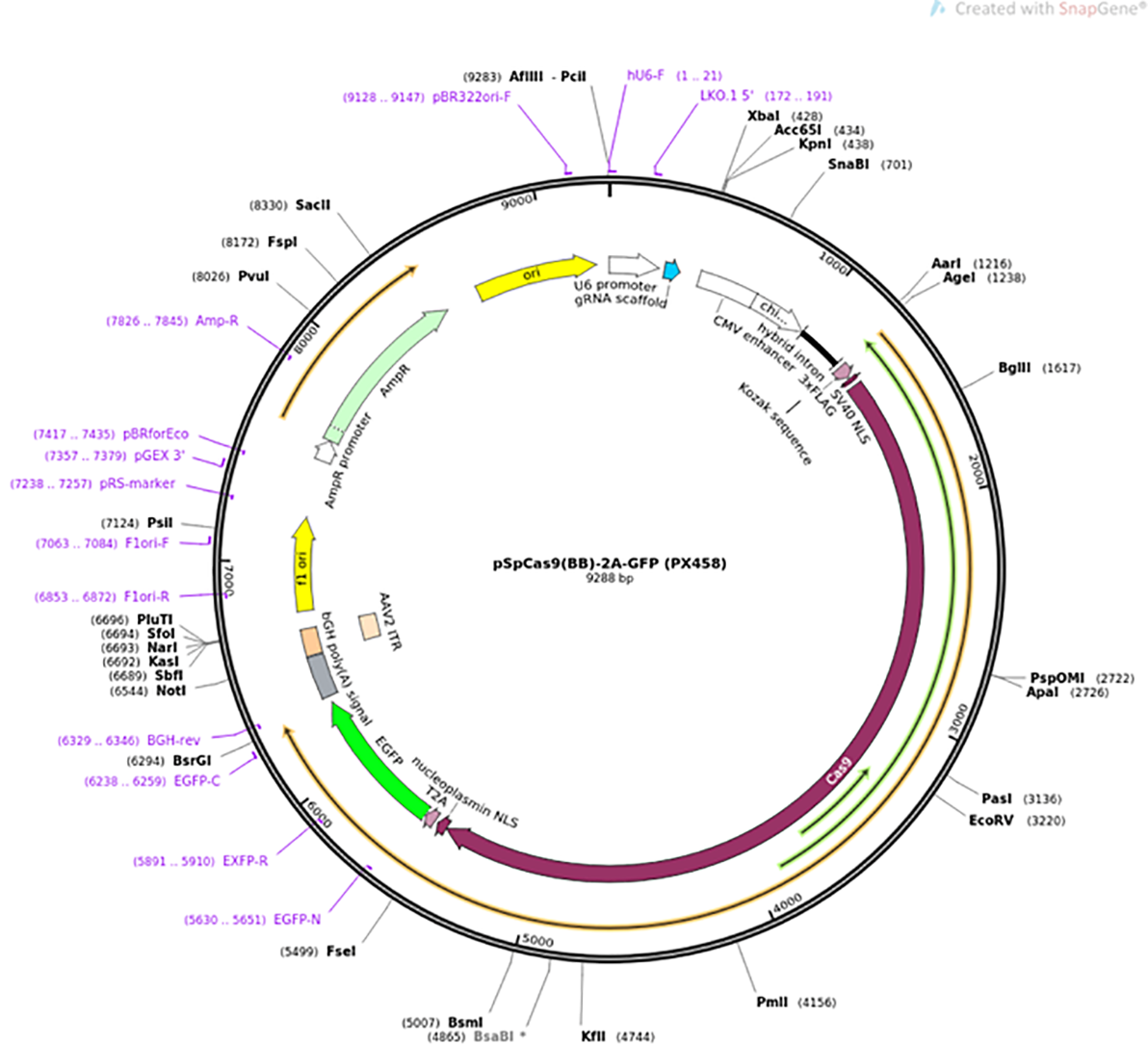


**Supplemental Figure 1: Plasmid map of pspCas9(BB)-2A-GFP plasmid.** Purchased from Addgene. Map freely available at company website.

| **Sequence cloned into pspCas9(BB)-2A-GFP plasmid** | **Sequence** |
| --- | --- |
| **Upstream sgRNA** | NNNNNNNNNNTCGNNNNCTTGGCTTTATATATCTTGTGGAAGGACGAAACACCGCGAAGGGCAAAATCTTCTAGGTTTNNNNGCTAGAAATAGCAAGTTAAAATAAGGCTAGTCCGTTATCAACTTGAAAAAGTGGCACCGAGTCGGTGCTTTTTTGTTTTAGAGCTAGAAATAGCAAGTTAAAATAAGGCTAGTCCGTTTTTAGCGCGTGCGCCAATTCTGCAGACAAATGGCTCTAGAGGTACCCGTTACATAACTTACGGTAAATGGCCCGCCTGGCTGACCGCCCAACGACCCCCGCCCATTGACGTCAATAGTAACGCCAATAGGGACTTTCCATTGACGTCAATGGGTGGAGTATTTACGGTAAACTGCCCACTTGGCAGTACATCAAGTGTATCATATGCCAAGTACGCCCCCTATTGACGTCAATGACGGTAAATGGCCCGCCTGGCATTGTGCCCAGTACATGACCTTATGGGACTTTCCTACTTGGCAGTACATCTACGTATTAGTCATCGCTATTACCATGGTCGAGGTGAGCCCCACGTTCTGCTTCACTCTCCCCATCTCCCCCCCCTCCCCACCCCCAATTTTGTATTTATTTATTTTTTAATTATTTTGTGCAGCGATGGGGGCGGGGGGGGGGGGN |
| **Downstream sgRNA** | NNNNNNNTTCGATNNCTTGGCTTTATATATCTTGTGGAAGGACGAAACACCGGGTTGCCTTGGGGAAAGTTAGTTTTAGAGCTAGAAATAGCAAGTTAAAATAAGGCTAGTCCGTTATCAACTTGAAAAAGTGGCACCGAGTCGGTGCTTTTTTGTTTTAGAGCTAGAAATAGCAAGTTAAAATAAGGCTAGTCCGTTTTTAGCGCGTGCGCCAATTCTGCAGACAAATGGCTCTAGAGGTACCCGTTACATAACTTACGGTAAATGGCCCGCCTGGCTGACCGCCCAACGACCCCCGCCCATTGACGTCAATAGTAACGCCAATAGGGACTTTCCATTGACGTCAATGGGTGGAGTATTTACGGTAAACTGCCCACTTGGCAGTACATCAAGTGTATCATATGCCAAGTACGCCCCCTATTGACGTCAATGACGGTAAATGGCCCGCCTGGCATTGTGCCCAGTACATGACCTTATGGGACTTTCCTACTTGGCAGTACATCTACGTATTAGTCATCGCTATTACCATGGTCGAGGTGAGCCCCACGTTCTGCTTCACTCTCCCCATCTCCCCCCCCTCCCCACCCCCAATTTTGTATTTATTTATTTTTTAATTATTTTGTGCAGCGATGGGGGCGGGGGGGGGGGGGGN |

**Supplemental Table 1: sgRNAs were cloned into pspCas9(BB)-2A-GFP plasmid in frame with Cas-9 plasmid.** Depicted are Sanger sequencing results of the pspCas9(BB)-2A-GFP plasmids after the sgRNA sequences targeting DNA upstream (top) and downstream (bottom) of the *CXADR* gene were cloned in. sgRNA sequences are highlighted. Sanger sequencing reactions were performed by Genewiz (South Plainfield, NJ).
